# Bioaccessibility of Organochlorine Pesticides in Contaminated Soil: A Poultry-Informed In Vitro Gastrointestinal Model

**DOI:** 10.3390/biology15161381

**Published:** 2026-08-13

**Authors:** Arailym Akhatzhanova, Farida Amutova, Moldir Nurseitova, Nurlan Akhmetsadykov, Yerbolat Sailaukhanuly, Gaukhar Konuspayeva, Stefan Jurjanz

**Affiliations:** 1 LLP “Scientific and Production Enterprise Antigen”, 4, Azerbayeva Str., Almaty 040905, Kazakhstannurseitova.moldyr@antigen.kz (M.N.);; 2 Biotechnology Department, Farabi University, 71 Al-Farabi Avenue, Almaty 050040, Kazakhstan; 3 Laboratory of Engineering Profile, Satbayev University, 22a Satpaev Str., Almaty 050013, Kazakhstan; 4 Laboratory Animal and AgroEcoSystems (L2A), University of Lorraine-INRAE, 54500 Vandoeuvre, France; stefan.jurjanz@univ-lorraine.fr

**Keywords:** organochlorine pesticides, bioaccessibility, Tenax, in vitro digestion, contaminated soil

## Abstract

Free-ranging poultry ingest 4–30 g of soil per day, making soil a significant route of organochlorine pesticide (OCP) exposure in agricultural areas with legacy contamination. However, total soil concentrations overestimate actual exposure. Only the bioaccessible fraction, mobilized within the gastrointestinal tract, governs transfer to eggs and meat. Static physiologically based extraction test (PBET) models consistently underestimate this fraction for hydrophobic compounds due to re-equilibration of desorbed contaminants onto the soil matrix. This study applied and evaluated a sink-modified static in vitro gastrointestinal model incorporating Tenax resin as a sorption sink to assess OCP bioaccessibility in spiked aged soil under simulated poultry digestive conditions. The aqueous phase-only fraction (gastric plus intestinal liquid) ranged from 0.004 to 17.1%, while the aqueous phase plus sorption sink (Tenax) fraction, measured from the same intestinal phase incubation, ranged from 24.0 to 52.7%, with the largest relative changes observed for HCH isomers (from near-undetectable levels without Tenax) and 2.7- to 4.3-fold increases for DDT-related compounds, a difference confirmed as statistically significant (paired *t*-test) for six of the seven compounds. One-way ANOVA revealed significant differences among compounds (F(6,21) = 5.53, *p* = 0.0014). Tukey’s HSD showed that β-HCH (56.1 ± 10.6%) and 4,4′-DDT (55.6 ± 24.0%) had significantly higher bioaccessibility than γ-HCH (25.8 ± 0.9%), 2,4′-DDD (22.2 ± 18.0%), and 4,4′-DDE (22.0 ± 5.7%), while α-HCH (28.6 ± 3.9%) and 4,4′-DDD (33.9 ± 8.1%) occupied an intermediate position, not significantly different from either group. A substantial fraction of mobilized OCPs was associated with the Tenax sink (17–53%), confirming that aqueous phase measurements alone underestimate the bioaccessible pool. These results demonstrate the importance of including a sorption sink in poultry-informed gastrointestinal models for accurate assessment of OCP transfer from contaminated soil to food products.

## Simple Summary

Free-ranging chickens often peck at soil while foraging and in areas where old pesticides were once stored or used; that soil can still contain harmful chemicals called organochlorine pesticides, even decades later. Not all of the pesticide in soil actually enters a chicken’s body: only the fraction that dissolves during digestion can be absorbed and later end up in eggs or meat. We built a laboratory model that mimics a chicken’s stomach and intestine to measure this “available” fraction accurately. Our model included a special resin that captures dissolved chemicals as they are released, mimicking how the body continuously absorbs them during digestion. We found that measuring the digestive fluid alone missed a substantial share of several pesticide compounds that the resin was able to capture, showing that fluid-only measurements likely underestimate the true amount released from soil. This approach offers a faster, lower-cost, and more animal-friendly way to assess pesticide exposure risk for poultry raised on contaminated land.

## 1. Introduction

Organochlorine pesticides (OCPs), including dichlorodiphenyldichloroethane (DDT) and its metabolites, hexachlorocyclohexane (HCH) isomers, and dieldrin, are persistent organic pollutants (POPs) listed under Annex A of the Stockholm Convention. Their combination of high hydrophobicity (log Kow ~ 3.7–6.9), resistance to metabolic and abiotic degradation, and strong sorption to soil organic matter results in environmental half-lives of decades [1]. These properties promote bioaccumulation in lipid-rich tissues and biomagnification along the food chain, with consequences including endocrine disruption, immunotoxicity, and carcinogenicity [2,3].

Central Asia, and Kazakhstan in particular, carries a significant legacy burden from Soviet-era agricultural intensification. Approximately 1500 tons of obsolete pesticide stockpiles, many classified as POPs, were identified across Kazakhstan in the early 2000s [4]. Residual OCP concentrations continue to be detected in soils, plants, and food in the Almaty region [5,6]. Abandoned and partly destroyed storage facilities have led to uncontrolled chemical dispersal into surrounding agricultural soils. This raises concern for both ecosystem integrity and human health through dietary exposure [7].

A critical but underexplored exposure pathway is the direct ingestion of contaminated soil by free-ranging poultry. Laying hens foraging on soil-contaminated ground ingest 4–30 g of dry soil per day depending on housing conditions [8,9], providing a substantial route for OCP uptake into the food chain. However, total OCP concentrations in soil do not adequately predict actual animal exposure. The bioaccessible fraction, defined as the proportion mobilized within the gastrointestinal (GI) tract and available for intestinal absorption, governs transfer to animal tissues [10,11].

Existing in vitro GI models for estimating contaminant bioaccessibility range from simple static physiologically based extraction test (PBET) approaches to complex dynamic systems. The two-phase (gastric-intestinal) model of Furman et al. [12] offers reproducibility and operational simplicity. However, like most static models, it does not account for continuous removal of mobilized contaminants. This leads to re-partitioning of desorbed compounds back onto the soil matrix. Dynamic models [13,14] more closely replicate avian physiology through mechanical action and fluid exchange. However, their experimental complexity limits routine applicability. In vitro models also offer ethical and logistical advantages over in vivo poultry studies, which require large numbers of animals, controlled dietary regimes, and tissue sampling. Critically, static PBET approaches have been shown to severely underestimate bioaccessibility of hydrophobic organic contaminants such as OCPs, with reported values below 4% for historically contaminated soils [15,16].

The inclusion of Tenax resin as a hydrophobic sorption sink has been proposed as a practical modification to overcome equilibrium limitations of static models. Acting as an infinite sink, Tenax adsorbs OCPs from the aqueous phase, continuously lowering their dissolved concentration. This disrupts the soil–water partitioning equilibrium and drives further desorption of OCPs from soil particles. The process mimics the role of bile salt micelles and intestinal epithelium, which similarly remove hydrophobic compounds from solution during absorption. This approach has yielded 3- to 22-fold increases in measured DDT bioaccessibility [17] and comparable enhancements for PAHs [18] and PBDEs [19]. Despite these advances, GI model applications in poultry research have remained largely restricted to nutrient digestibility, feed enzyme efficacy, and feed additive performance [2]. Contaminant-focused studies have been limited to mycotoxin sequestration by dietary sorbents [20] and heavy metal bioaccessibility [21]. To our knowledge, no study has applied a poultry-informed GI model to assess OCP bioaccessibility. The fraction of soil-bound OCPs available for intestinal absorption and transferable to eggs and meat therefore remains unknown. This limits the accuracy of risk assessments and mitigation strategies for contaminated agricultural areas.

This study applied and evaluated a sink-modified static in vitro GI model incorporating Tenax as a sorption sink. It was used to assess OCP bioaccessibility in contaminated soil under simulated poultry gastrointestinal conditions. We hypothesized that Tenax inclusion would significantly increase bioaccessibility relative to conventional aqueous phase PBET. We also hypothesized that bioaccessibility would differ among OCP congeners depending on their physicochemical properties and soil sorption behavior.

## 2. Materials and Methods

### 2.1. Materials and Reagents

Pepsin from porcine gastric mucosa (400 units/mg protein), pancreatin from porcine pancreas (8 × USP), Tenax Porous Polymer Adsorbent (80–100 mesh), n-hexane, anhydrous magnesium sulfate, sodium chloride, dichloromethane, acetone were from Sigma-Aldrich (Sigma-Aldrich, St. Louis, MO, USA). Standards of certified OCPs: DDTs (2,4′-DDD, 4,4′-DDD, 4,4′-DDT, 4,4′-DDE) and HCH (α-HCH, β-HCH, γ-HCH) were purchased from Lezart LTD (Almaty, Kazakhstan); Hexachlorobenzene 13C6 used as an internal standard was obtained from Dr. Erhenstorfer (LGC Standards, Augsburg, Germany).

### 2.2. Soil Spiking and Aging

The soil samples were collected from a garden (43.169472° N, 76.768687° E) in the Almaty region (Kazakhstan) and analyzed for the presence of OCPs. After confirming that the soil was free of OCP contaminations (<LOQ, which is 0.08 µg/g), its physicochemical properties, including moisture content, pH, dry matter, ash content and organic matter content were determined. The soil was subsequently air-dried at room temperature and passed through a 2 mm sieve.

To prepare contaminated soil, an OCP standard stock solution (10 mg/L) was added to soil to achieve a final concentration of 0.5 µg/g of dry soil. The spiked soil was aged for approximately six months in closed glass containers under dark conditions. The soil was periodically mixed during the aging period. The samples were stored in a roofed, unheated attic space exposed to ambient environmental conditions; therefore, temperature and humidity were not controlled and varied according to outdoor weather conditions, ranging from below 10 °C in winter to approximately 20 °C in spring. No moisture was added during aging.

### 2.3. In Vitro Digestion Simulation

**Figure 1:**
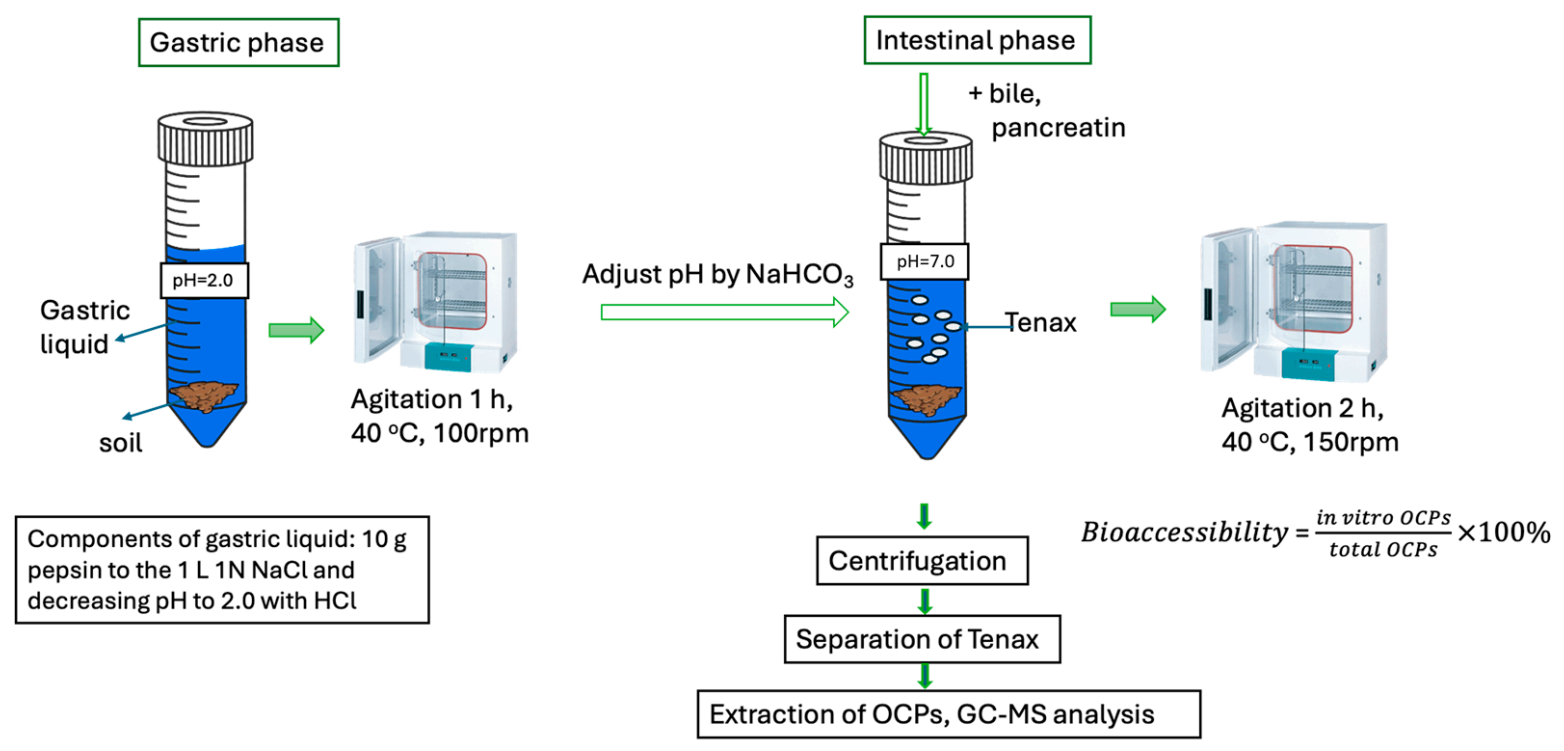
Schematic diagram of the sink-modified static in vitro gastrointestinal model for assessment of OCP bioaccessibility in soil under simulated poultry digestive conditions.

A variety of in vitro digestion simulation methods have been developed to estimate the bioaccessibility of pollutants. In this study an in vitro digestion model described elsewhere with some modifications [12] was used to assess the OCP bioaccessibility in soil with the inclusion of a sorption sink. Tenax was used as a sorption sink. Before use, Tenax (80–100 mesh) was wrapped with filter paper and was extracted three times with hexane:acetone (*v*/*v* 1:1) in an ultrasonic bath for 5 min. The gastric phase was prepared by addition of 10 g pepsin to the 1 L 1N NaCl and decreasing pH to 2.0 with HCl. Briefly, 1 g of dry soil was added to the gastric fluid (20 mL) in 50 mL Falcon tubes and incubated for 1 h at 40 °C and 150 rpm. Following the gastric phase incubation, gastric fluid was modified to the intestinal phase by gradually raising the pH to 7.0 via dropwise addition of 1.0 M NaHCO_3_ and adding 0.01 g of pancreatin and 0.035 g of bile salts; also 0.2 g of Tenax was added as an absorptive sink to the tube as mentioned elsewhere [17]. The amount of Tenax used (0.2 g) was informed by Li et al. [17], who demonstrated that Tenax added at 0.01–0.2 g to the PBET intestinal solution sorbed approximately 100% of DDT and its metabolites within 6.3–19 min, confirming its effectiveness as a saturating sorption sink. Given the organic carbon (OC) content of the soil used in this study (1.95%), the resulting Tenax:OC ratio was approximately 10.3:1, comfortably exceeding the minimum ratio of 5:1 recommended for effective Tenax-based extraction of hydrophobic organic contaminants [22], supporting that 0.2 g of Tenax provided an adequate sorption sink under the present experimental conditions. The intestinal phase continued to be incubated for 2 h at 40 °C under 150 rpm. After incubation Tenax was collected, and supernatant and soil were separated by centrifugation at 5000 rpm for 10 min (Figure 1).

OCP bioaccessibility was calculated using the following equation:

$$\mathrm{Bioaccessibility}\;=\;\frac{\mathrm{in}\;\mathrm{vitro}\;\mathrm{OCPs}}{\mathrm{total}\;\mathrm{OCPs}}\times \;100\%$$

where in vitro OCPs is the mass of OCPs extracted by the in vitro method, and total OCPs is the mass of OCPs in soil prior to extraction.

### 2.4. Sample Extraction

After centrifugation OCPs extraction was conducted from supernatant, Tenax and residual soil separately. OCPs were extracted from supernatant by liquid–liquid extraction (LLE) [23]. The sample of supernatant (10 mL) was extracted by 15 mL of dichloromethane:hexane (1:1) and vortexed for 3 min. Then the samples were centrifuged for 5 min at 5000 rpm. The supernatant passed through filter paper with anhydrous magnesium sulfate to a round-bottom flask. The extraction was repeated twice. The combined filtrate was concentrated at 60 °C by a rotary evaporator (IKA-Werke GmbH & Co. KG, Staufen, Germany) to approximately 1.5 mL.

Tenax was dried and extracted with 10 mL of acetone by ultrasonic bath 3 times. All extracts were combined and evaporated to approximately 1.5 mL.

The residue soil samples were extracted by the QuEChERS method as described by Pinto et al. [24] with slight modifications. To the residue soil samples 1.5 mL of deionized water was added and was shaken for 1 min with a Vortex (IKA-Werke GmbH & Co. KG, Staufen, Germany). Then, 2.5 mL of ethyl acetate was added, and the mixture was shaken for 5 min again. Following this, 1 g of anhydrous MgSO_4_ was added and shaken as quickly as possible to prevent the formation of MgSO_4_ conglomerates. The tubes were then centrifuged at 5000 rpm for 5 min, and the upper layer was collected for the following GC-MS analysis.

### 2.5. Sample Analysis

The analysis of the extracts was performed by Gas Chromatography–Mass Spectrometry (GC-MS) (7890 B, Agilent Technologies, Santa Clara, CA, USA). Briefly, the mass spectrometer was set to a resolution of 10,000 in the electron ionization mode (70 eV electron energy) (5975C, Agilent Technologies, Santa Clara, CA, USA). An HP-5ms Ultra Inert capillary column (30 m × 0.25 mm × 0.25 μm) from Agilent J&W (Agilent Technologies) in splitless mode was used. The GC temperature program for OCP analysis was: from 120 °C (1 min), 40 °C min^−1^ to 220 °C (13 min). The total time was 16.5 min. The obtained signals were integrated using the Mass Hunter program (B 07.05.2479).

### 2.6. Quality Control

An analysis of in vitro digestion simulation was performed in four independent trials (*n* = 4). Blank samples were included in each trial. Quantification was carried out using an internal standard (^13^C-labeled HCB). The recovery of spiked soil samples, as well as the limits of detection (LOD) and quantification (LOQ), are presented in Table 1 and Table 2.

### 2.7. Statistical Treatment

Statistical analysis was performed using one-way ANOVA to evaluate differences in mean bioaccessibility of OCPs among gastrointestinal phases, followed by Tukey’s HSD test for multiple comparisons. Statistical significance was defined at the 95% confidence level (*p* < 0.05) (XLSTAT version 2023.1.3.1407).

## 3. Results and Discussion

### 3.1. Soil Characterization

Prior to bioaccessibility assessment, the physicochemical properties of the soil used in this study were determined, as these parameters are known to exert a considerable influence on the sorption behavior, persistence, and ultimately the bioaccessibility of organochlorine pesticides (OCPs) in the soil matrix [25].

Soil moisture content was 8.1%, indicating a relatively low water content typical of an air-dried state. Dry matter content was 92.5%, confirming that the samples consisted predominantly of solid material. Moisture content is a critical parameter in soil characterization, as it governs the activity of pore water, influences pollutant partitioning between the aqueous and solid phases, and affects the accessibility of contaminants [26].

Ash content was determined by ignition at 550 °C following standard protocols [27] and was 27.9% expressed on a fresh matter basis. This value is relative to fresh weight and accounts for residual moisture [28]. The mass fraction of organic carbon was 1.95%, corresponding to an organic matter content of approximately 3.4%, calculated using the van Bemmelen conversion factor of 1.724 [29]. This organic matter level is consistent with moderately organic soils. Organic carbon content is a primary driver of sorption and retention of hydrophobic organic pollutants through hydrophobic partitioning into the soil organic phase [1].

The soil pH was 8.0, indicating slightly alkaline conditions. Soil pH is a key factor governing the mobility and bioavailability of heavy metals and ionic contaminants [30]. OCPs are predominantly non-ionic and hydrophobic. Their environmental behavior is largely independent of pH. Their fate is governed primarily by organic matter content, soil texture, and temperature [1,31].

The mean OCP concentrations in the spiked aged soil ranged from 0.46 to 0.50 µg/g dry weight (Table 2), consistent with the nominal spiking level of 0.5 µg/g. Because the moderate organic matter content (3.4%) limits the sorptive capacity of the soil matrix, it may facilitate contaminant mobilization during simulated digestion relative to soils with higher organic matter content [1]. Recovery rates ranging from 92% to 100% confirmed the accuracy of the extraction and quantification procedures [32].

### 3.2. The Bioaccessibility of OCPs In Vitro Gastrointestinal Model of Poultry

**Figure 2:**
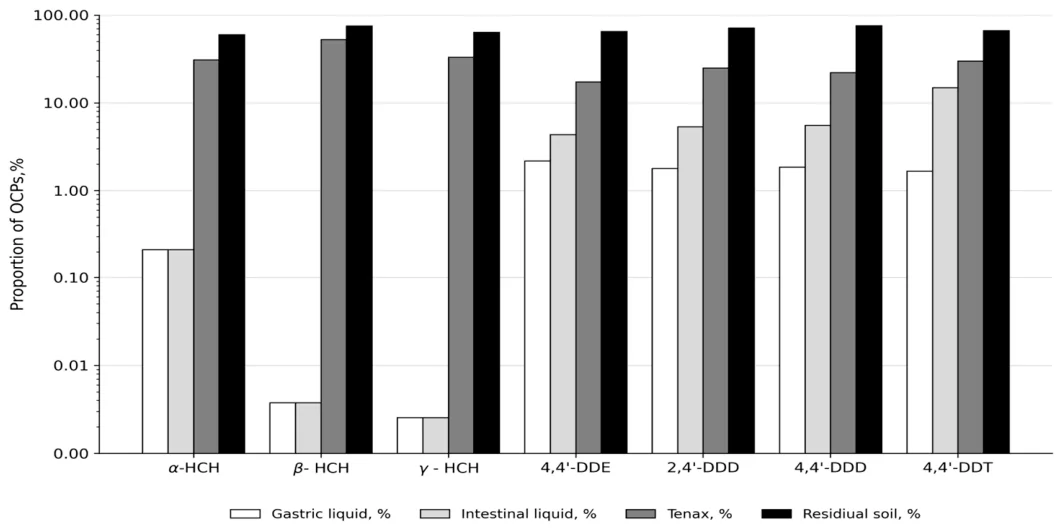
Distribution of OCPs among gastrointestinal phases and residual soil, %.

The observed increase in OCP mobilization from gastric to intestinal conditions in this study is consistent with previous findings for hydrophobic organic contaminants [33,34,35,36]. While OCP bioaccessibility in the gastric phase was virtually non-existent (e.g., 4,4′-DDT: 1%), a substantial increase was observed in simulated intestinal conditions (e.g., up to 16%), reflecting the enhanced mobilization capacity of intestinal fluids. The distribution of OCPs between the simulated gastrointestinal phases, Tenax and residual soil (%) is presented on a logarithmic scale in Figure 2.

Similar trends have been widely reported for other hydrophobic contaminants, including BDE-209 [37], PCBs [34,38], and PAHs [35], where bioaccessibility in intestinal conditions significantly exceeded that in gastric conditions. Tang et al. [35] observed that PAH bioaccessibility was significantly higher in the small intestine (up to 60.5%) than in the gastric phase (up to 54.9%), with intestinal-to-gastric ratios reaching 9.7. This effect is attributed to bile salts, which act as natural surfactants and promote the formation of mixed micelles [34,38].

Although OCPs are non-ionic and their behavior is largely pH-independent, the pH increase during the intestinal phase may indirectly promote contaminant mobilization through enhanced solubility of soil organic matter [35].

The present results show that aqueous phase measurements alone underestimate the available fraction of OCPs. A substantial portion of mobilized contaminants was associated with the Tenax fraction (17–53%). This confirms the importance of including a sorption sink to capture the full bioaccessible pool. Tao et al. [36] reported similar findings, showing that a significant fraction of mobilized OCPs remains associated with non-aqueous phases when no sink is present. The predominance of OCPs in the residual soil fraction (59–78%) confirms strong sorption to soil organic matter, consistent with the known hydrophobicity of these compounds.

**Figure 3:**
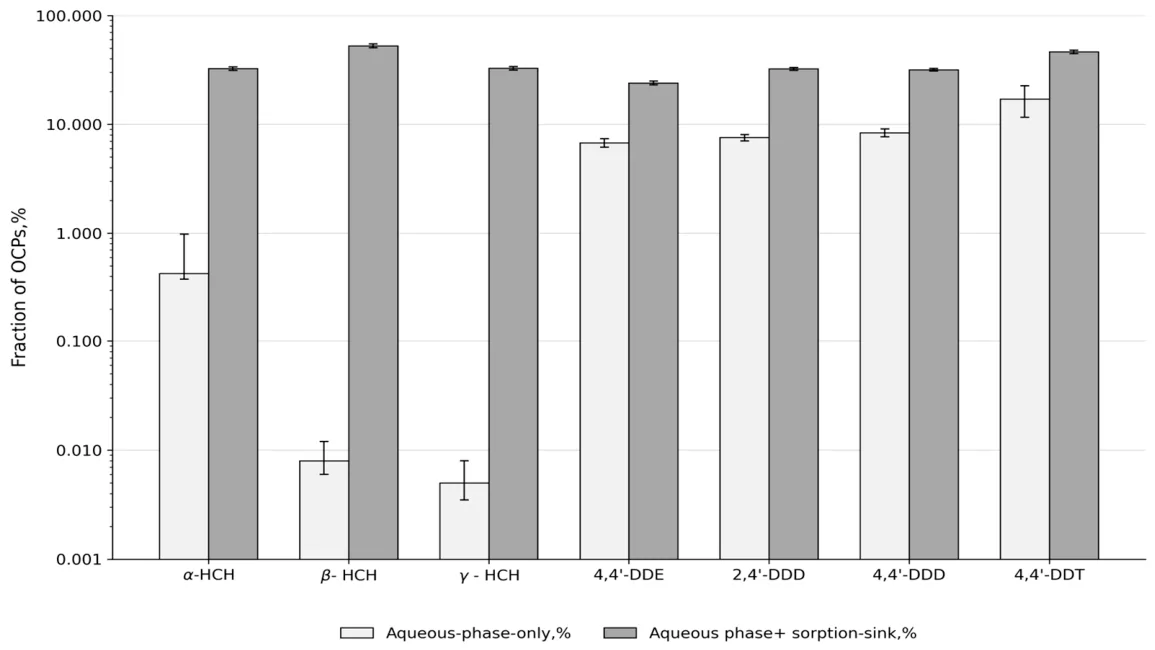
Aqueous phase-only fraction versus aqueous phase plus sorption sink (Tenax) fraction of OCPs.

The substantially larger sink-captured fraction relative to the aqueous phase alone, observed in this study, is consistent with previous research demonstrating the critical role of sorption sinks in overcoming limitations of static in vitro models. In the present work, the aqueous phase-only fraction ranged from 0.004 to 17.1%, while the aqueous phase plus sorption sink fraction, measured from the same intestinal phase incubation, ranged from 24.0 to 52.7% (Figure 3, logarithmic scale). This corresponds to several-fold enhancements for DDT-related compounds (2.7- to 4.3-fold) and a shift from near-undetectable to substantial bioaccessibility for HCH isomers, whose aqueous phase concentrations without Tenax were at or below the limit of detection. A paired *t*-test (trial-level values, *n* = 4) confirmed a statistically significant increase in the aqueous + sorption sink fraction relative to the aqueous phase-only fraction for six of the seven compounds (*p* < 0.05); the increase for 2,4′-DDD was not statistically significant (*p* = 0.271), consistent with its high inter-trial variability. Similar trends have been widely reported: a 3.4–22-fold increase in DDTr bioaccessibility was observed using TI-PBET [17], and average enhancements of up to 9-fold were reported across different in vitro methods like PBET, DIN, IVD [39]. Other studies have shown comparable effects, including 4.4-fold increases for PAHs [18] and up to 8-fold for PBDEs [19] when Tenax was applied as a lipophilic sink. Likewise, inclusion of a sorption phase (silicone cord) increased DDTr desorption by up to 20-fold, highlighting that conventional PBET approaches substantially underestimate bioaccessibility due to insufficient capacity for hydrophobic organic contaminants [15].

The underlying mechanism is the ability of Tenax to act as an infinite sink, continuously removing desorbed contaminants from the aqueous phase and maintaining a concentration gradient that promotes further desorption from the soil matrix. Without such a sink, equilibrium conditions limit contaminant release. This leads to artificially low bioaccessibility values. Several studies reported DDTr bioaccessibility below 4% in historically contaminated soils when no sink was used [15,16]. The magnitude of Tenax-induced enhancement may vary depending on model composition. Li et al. [40] reported increases of up to 86% using an unfed batch method (UBM). Smaller differences were observed in more complex systems such as FOREhST, likely due to interactions with food components.

**Figure 4:**
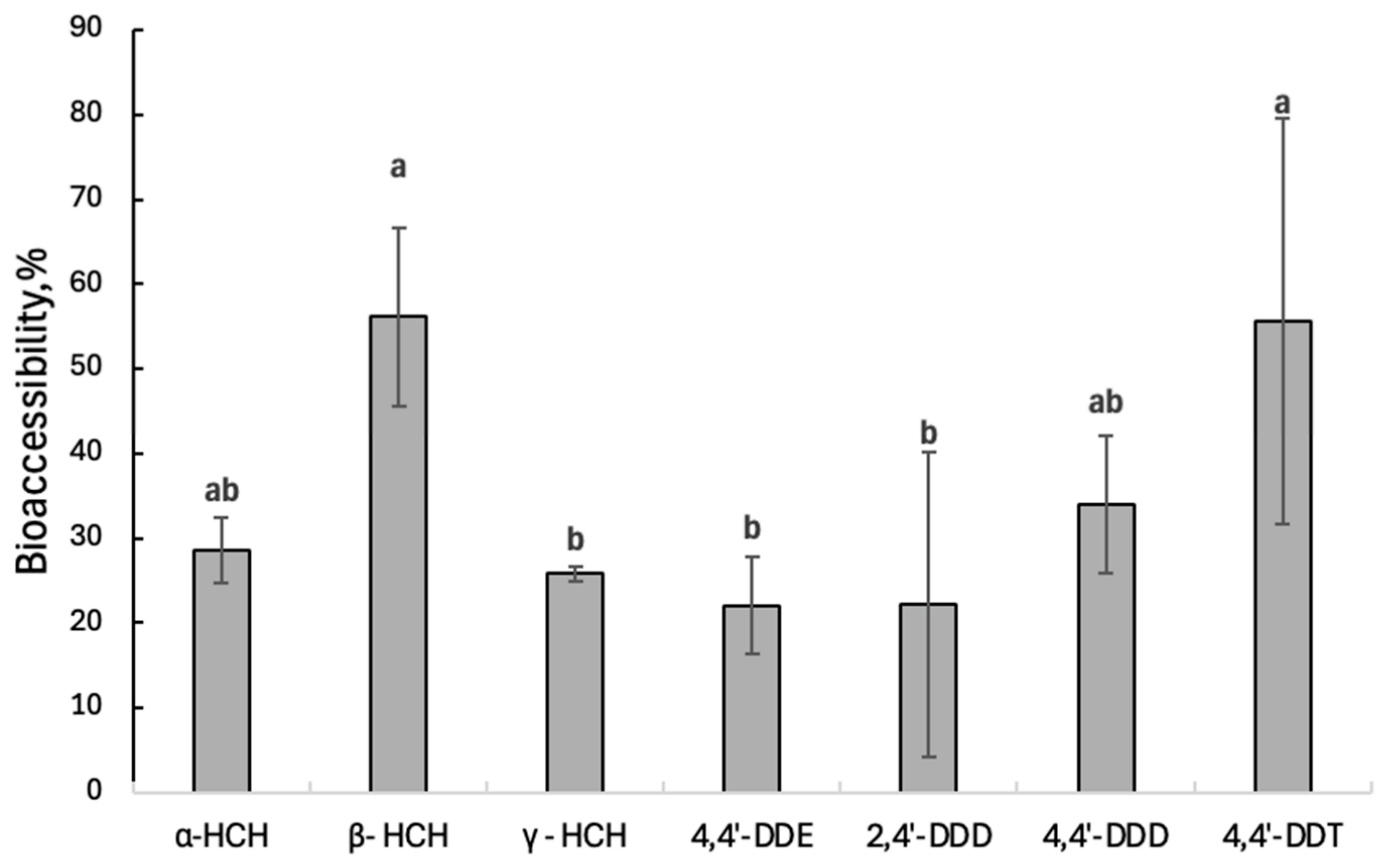
Mean bioaccessibility (%) of OCPs obtained using the in vitro digestion method. Error bars represent standard deviation (*n* = 4 independent trials). Bars sharing the same letter (a, ab, or b) are not significantly different from one another (Tukey’s HSD, *p* > 0.05); bars with different letters differ significantly (*p* < 0.05).

The mean bioaccessibility of OCPs differed significantly among compounds (one-way ANOVA on trial-level means, *n* = 4 independent trials per compound: F(6,21) = 5.53, *p* = 0.0014). Tukey’s HSD post hoc test showed that β-HCH (56.1 ± 10.6%) and 4,4′-DDT (55.6 ± 24.0%) had significantly higher bioaccessibility than γ-HCH (25.8 ± 0.9%), 2,4′-DDD (22.2 ± 18.0%), and 4,4′-DDE (22.0 ± 5.7%) (*p* < 0.05 for all six pairwise comparisons). α-HCH (28.6 ± 3.9%) and 4,4′-DDD (33.9 ± 8.1%) did not differ significantly from either the higher- or lower-bioaccessibility compounds (*p* > 0.05 in all cases), occupying an intermediate position (Figure 4). All seven compounds were quantifiable above their respective limits of detection/quantification when summed across all gastrointestinal fractions (liquid, Tenax, and residual soil) in all four independent trials, although α-, β-, and γ-HCH were individually below the limit of detection in the aqueous gastric and intestinal phases alone. Notably, 4,4′-DDT and 2,4′-DDD showed substantially higher inter-trial variability (SD = 24.0% and 18.0%, respectively) than the other compounds, driven primarily by consistently lower bioaccessibility values observed in one trial across several compounds; this pattern is discussed further as a study limitation below.

Notably, one trial consistently yielded lower bioaccessibility values relative to the other three trials for several compounds, most prominently 4,4′-DDT and 2,4′-DDD. A formal outlier assessment (Grubbs’ test) identified the corresponding 4,4′-DDT value as a borderline statistical outlier, though the test’s power is limited at this sample size (*n* = 4) and no other trial-level value met the criterion for exclusion. Given the limited power to confidently distinguish a genuine outlier from natural experimental variability at this sample size, all data points were retained in the reported analysis rather than excluded on a case-by-case basis. We cannot rule out an unidentified procedural deviation in this trial; however, in the absence of documented evidence of such a deviation, an exclusion was not statistically or methodologically justified. This trial-to-trial variability, particularly for 4,4′-DDT and 2,4′-DDD, is acknowledged as a limitation of the current dataset and underscores the value of increasing the number of independent trials in future work to improve the precision of bioaccessibility estimates for these compounds. Additional limitations include the absence of a soil-free control to independently verify background contamination and Tenax extraction efficiency in the absence of soil.

The intermediate, statistically overlapping position of α-HCH and 4,4′-DDD is consistent with their physicochemical properties lying between those of the high-bioaccessibility (β-HCH, 4,4′-DDT) and low-bioaccessibility (γ-HCH, 2,4′-DDD, 4,4′-DDE) compounds. Rather than reflecting distinct discrete categories, these results suggest that OCP bioaccessibility in aged soil follows a continuum shaped by compound-specific sorption behavior (log Kow, stereochemistry, degree of aging-related sequestration), with β-HCH and 4,4′-DDT representing the upper end of this continuum under Tenax-enhanced conditions.

The higher bioaccessibility of 4,4′-DDT compared to its degradation products 4,4′-DDE and 4,4′-DDD is consistent with compound-specific sorption during aging. DDT metabolites, particularly 4,4′-DDE, have higher log Kow values (≈6.5–6.8) and therefore undergo stronger hydrophobic partitioning into soil organic matter over time, reducing their desorption during simulated digestion [1,15]. Among HCH isomers, β-HCH showed higher bioaccessibility than α-HCH and γ-HCH. This reflects its distinct stereochemistry: the all-axial conformation of chlorine substituents in β-HCH promotes mobilization in the presence of bile salt micelles [34]. Higher variability for 4,4′-DDT (SD = 24.0%) and 2,4′-DDD (SD = 18.0%) likely reflects sensitivity to minor differences in soil organic matter microstructure.

Once absorbed across the intestinal epithelium, OCPs—being highly lipophilic (log Kow: DDT ≈ 6.9, DDE ≈ 6.5, DDD ≈ 6.0)—are expected to distribute preferentially into lipid-rich compartments and be transferred into developing egg yolk via passive diffusion across the oocyte membrane and/or association with yolk precursor lipoproteins (very-low-density lipoprotein and vitellogenin) synthesized in the liver and transported to the ovary during follicle development [41,42]. In laying hens specifically, Furusawa et al. [43] demonstrated that orally administered p,p′-DDT is rapidly metabolized to p,p′-DDE and p,p′-DDD and distributed to egg-forming tissues (liver, ovary, oviduct) and egg yolk within 24 h, with DDT and DDE reaching the yolk while DDD remained largely confined to the liver. This differential tissue distribution among DDT and its metabolites suggests that bioaccessibility estimates alone are insufficient to predict egg contamination risk; compound-specific toxicokinetics, including metabolic transformation and tissue-specific retention, must also be considered when translating bioaccessibility into an egg transfer risk estimate.

The bioaccessibility values in this study (22–56%) are within the range reported for hydrophobic organic contaminants. Reported values vary widely by compound class and experimental design: from <4% for DDTr [15,16] to 5–61% for PCBs [44] and to as high as 91% for PAHs [45]. The higher values obtained here for 4,4′-DDT and β-HCH are attributed to the Tenax sorption sink, which enhances desorption and prevents re-equilibration of contaminants onto the soil matrix.

The incubation times used (1 h gastric, 2 h intestinal) are consistent with PBET-based approaches and are sufficient to approach equilibrium, since most desorption occurs within the initial stages of digestion [12]. Bioaccessibility in this study is therefore governed primarily by sorption–desorption processes and micelle formation, rather than by kinetic limitations.

The inclusion of Tenax in in vitro digestion models has also been shown to improve the predictive capability of bioaccessibility assays with respect to in vivo bioavailability. The incorporation of Tenax significantly enhanced in vivo–in vitro correlations. For the TI-PBET method, r^2^ increased from 0.01 to 0.37. An acceptable correlation was also achieved for the TI-DIN method (r^2^ = 0.66; slope = 0.78 ± 0.21) [44]. These findings indicate that sorption sinks improve the physiological relevance of in vitro models. However, the effect was method dependent. Only marginal improvement was observed for TI-IVD, suggesting that model composition influences the effectiveness of Tenax inclusion.

The observed differences in bioaccessibility among DDT and its metabolites can be further understood in the context of aging-related sequestration. With increasing soil residence time, DDT residues progressively redistribute from readily desorbable to more strongly bound sorption sites through slow diffusion into soil organic matter micropores and, potentially, covalent interactions with organic matter—a process broadly termed bound residue formation. Because this sequestration process is governed in part by compound hydrophobicity, more hydrophobic compounds are expected to become progressively less accessible with aging relative to their less hydrophobic metabolites. Consistent with this, Taylor et al. [46] reported that Tenax-based bioaccessibility of aged DDT derivatives in marine sediment decreased in the order DDD > DDE > DDT, negatively correlated with log Kow, reflecting greater aging-related sequestration of the more hydrophobic parent compound relative to its metabolites. This pattern offers a plausible explanation for compound-specific differences observed in our aged soil.

The moderately low organic carbon content of the soil used in this study (1.95% OC; ~3.4% organic matter) is expected to influence the generalizability of our bioaccessibility estimates to soils with different OC content. Because OCP sorption to soil is governed primarily by partitioning to organic matter (Kd = Koc × Foc), soils with higher OC content would be expected to exhibit stronger sorption and sequestration of OCPs, potentially resulting in lower bioaccessibility due to reduced desorption into the digestive fluid during the simulated gastrointestinal transit time. Conversely, soils with lower OC content would be expected to show comparatively higher bioaccessibility. Our Tenax-to-organic carbon ratio (approximately 10.3:1) exceeded the recommended minimum of 5:1 for effective sink performance [22]; however, in soils with substantially higher OC content, a proportionally greater Tenax mass would likely be required to maintain an equivalent sink capacity. We recommend that future applications of this model to soils spanning a wider range of OC content include a corresponding adjustment of Tenax dosing based on the Tenax:OC ratio, rather than a fixed Tenax mass.

Previous studies also highlight that food components and digestive enzymes can further affect model performance. A fasted-state PBET was insufficient for predicting the relative bioavailability (RBA) of DDTr. The inclusion of lipase significantly improved the correlation (r^2^ = 0.66; slope = 1.16 ± 0.31) [47]. These findings indicate that Tenax enhances desorption-driven processes. However, accurate prediction of in vivo bioavailability requires consideration of both sorption dynamics and biochemical digestion processes.

Although direct in vivo validation for the specific OCPs examined here is not available, in vivo studies in laying hens using structurally related legacy POPs offer indirect context. Fournier et al. [11] and Jondreville et al. [8] both reported near-complete relative bioavailability (RBA ≈ 1) of soil-bound PCBs and chlordecone, respectively, in laying hens—considerably higher than the Tenax-enhanced in vitro bioaccessibility estimates reported here (24.0–52.7%). This discrepancy may reflect several factors: (1) differences in soil-binding behavior between PCBs/chlordecone and the OCPs examined in this study; (2) the longer in vivo gastrointestinal retention time and continuous absorption/re-circulation processes (e.g., enterohepatic recycling) in live animals, which cannot be fully replicated in a static 3 h in vitro assay even with a sorption sink; and (3) possible underestimation by our Tenax-based model despite its improvement over conventional static PBET. This comparison underscores that Tenax-enhanced in vitro bioaccessibility, while a substantial improvement over static aqueous phase-only estimates, may still represent a conservative (lower-bound) proxy for true in vivo bioavailability in poultry and highlights the need for direct in vivo validation of this specific model with OCPs in future work.

Compared to in vivo relative bioavailability trials, which require housing, dosing, and dissection of live animals over multi-week exposure periods, Tenax-modified in vitro bioaccessibility assays are substantially faster, lower-cost, and avoid ethical constraints associated with animal testing, making them more suitable for routine or large-scale screening applications [48]. This cost advantage is particularly relevant for site-specific risk assessment across the numerous legacy pesticide storage and disposal sites present in Kazakhstan and Central Asia, where in vivo testing at each site would be logistically and financially prohibitive. However, this efficiency comes with the trade-off of requiring further in vivo validation, as discussed above, before Tenax-modified bioaccessibility values can be used as direct substitutes for measured bioavailability in quantitative risk assessment.

The substantially larger sink-captured fraction relative to the aqueous phase alone supports the role of the sorption sink in enhancing the biological relevance of the in vitro model, although further validation against in vivo data remains necessary.

Multiple studies report chicken duodenal/small intestinal pH in the range of 5.7–6.6 [49,50,51,52], and the avian-specific model of Furman et al. [12] used an intestinal phase pH of 6.2. Our use of pH 7.0 was adopted from the broader mammalian PBET tradition rather than derived specifically from avian data, and we recognize this as a limitation of the current protocol. We recommend that future iterations of this model adopt pH 6.0–6.5 for the intestinal phase to more closely reflect avian physiology. The gastric/gizzard phase pH (2.0) is well within the physiological range reported for chicken gizzard (approximately 1.2–4.2) and consistent with Furman et al. [12] (2.0–3.2). The incubation temperature used (40 °C) is reasonably close to the reported core body temperature of chickens (approximately 40–42 °C) [53]. Porcine pepsin/pancreatin were used as standard surrogates due to the lack of commercially available avian-specific enzyme preparations, consistent with common practice in the poultry in vitro digestion literature. Avian bile acid composition differs substantially from mammalian bile—chicken bile is predominantly taurine-conjugated, dominated by taurochenodeoxycholic acid (up to ~85% of the bile acid pool)—whereas the commercial bile extract used here is compositionally distinct [54], representing a further limitation acknowledged here.

Because Tenax was introduced only during the intestinal phase, consistent with the TI-PBET protocol of Li et al. [17], we cannot rule out that a fraction of OCPs desorbed during the 1 h gastric phase re-adsorbed onto the soil matrix during the subsequent pH transition, prior to Tenax capture. This would result in a conservative (i.e., underestimated) bioaccessibility value, since re-adsorbed contaminants would need to re-desorb during the intestinal phase to be captured by Tenax. Future work directly comparing gastric-phase and intestinal-only Tenax inclusion would help quantify the magnitude of this potential artifact for OCPs specifically.

A limitation of this design is the absence of a fully independent Tenax-free intestinal phase control. Because the aqueous phase-only fraction reported here was measured from the same tube as the sorption sink fraction, rather than from a parallel Tenax-free incubation, a true no-sink aqueous equilibrium concentration—which would likely be higher, since no sink was continuously removing desorbed compounds—was not directly measured. Future work should include an independent Tenax-free digestion series to directly quantify the magnitude of Tenax-induced enhancement relative to a true no-sink baseline.

Although the pH transition from 2.0 to 7.0 was performed gradually via dropwise NaHCO_3_ titration, consistent with standard PBET methodology [55], this transition (completed within several minutes) remains more rapid than the corresponding in vivo process, which occurs progressively over a longer period as gastric contents are released into the small intestine. Because Tenax is introduced concurrently with the pH adjustment, the intestinal phase sink should begin capturing desorbed compounds promptly following the transition, likely limiting the extent of any resulting artifact.

## 4. Conclusions

This study demonstrates that incorporating Tenax as a sorption sink into a static in vitro digestion model improves the assessment of OCP bioaccessibility in contaminated soils. Conventional PBET approaches underestimate bioaccessibility due to equilibrium limitations, whereas Tenax enables continuous removal of desorbed contaminants, better simulating intestinal absorption under non-equilibrium conditions.

OCP bioaccessibility was compound-specific, with mean values ranging from 22% to 56%, and increased significantly in the presence of Tenax. Because a substantial fraction of mobilized contaminants was captured by the sorption sink rather than remaining in the aqueous phase, aqueous phase measurements alone are insufficient to estimate the bioaccessible pool, confirming that sorption–desorption processes govern contaminant availability in the gastrointestinal environment.

The developed approach bridges the gap between simple static and complex dynamic models, improving physiological relevance while maintaining experimental simplicity. The results are consistent with the literature data, supporting the robustness of the method. This study also contributes to addressing a research gap by applying a poultry-informed in vitro model to assess the bioaccessibility of persistent organic pollutants in a poultry context.

Future work should validate the model against in vivo data. Incorporation of additional physiological parameters, such as dietary components and enzymatic activity, would further improve predictive accuracy, and the proposed method offers a valuable tool for environmental risk assessment while also contributing to reducing reliance on animal testing in contaminant transfer studies.

## Figures and Tables

**Table 1:** Data for OCPs. Based on a typical concentration range of 0.025–0.5 ppm with 5 calibration points.

Compound	Qualifying Ions *m*/*z*	Retention Time, min	Calibration Equation	Determination Coefficient R^2^
α-HCH	183	4.9	y = 18.183x	0.9997
β-HCH	183	5.2	y = 13.976x	0.999
γ-HCH	183	5.3	y = 14.888x	0.9994
4,4′-DDE	246	9.4	y = 27.545x	0.9986
2,4′-DDD	235	9.7	y = 35.412x	0.9986
4,4′-DDD	235	11.1	y = 31.976x	0.9992
4,4′-DDT	235	13.1	y = 17.39x	0.9997
^13^C HCB *	255	5.0	-	-

* Internal standard.

**Table 2:** Extraction of organochlorine pesticides by QuEChERS from aged soil (0.5 µg g^−1^).

Compounds	Mean Concentration in Aged Soil ± SD, µg g^−1^	Recovery, %	LOD, µg g^−1^	LOQ, µg g^−1^
α-HCH	0.46 ± 0.01	92	0.005	0.01
β-HCH	0.50 ± 0.04	100	0.019	0.06
γ-HCH	0.49 ± 0.05	99	0.021	0.06
4,4′-DDE	0.46 ± 0.03	92	0.009	0.03
2,4′-DDD	0.5 ± 0.06	99	0.012	0.04
4,4′-DDD	0.46 ± 0.1	92	0.023	0.07
4,4′-DDT	0.45 ± 0.12	90	0.051	0.15

## Data Availability

The data that support the findings of this study are available from the corresponding author upon reasonable request.

## Abbreviations

The following abbreviations are used in this manuscript:

OCP	Organochlorine pesticide
PBET	Physiologically based extraction test
DDT	Dichlorodiphenyldichloroethane
HCH	Hexachlorocyclohexane
POPs	Persistent organic pollutants
PAH	Polycyclic aromatic hydrocarbon
PBDE	Polybrominated diphenyl ethers
GC-MS	Gas Chromatography–Mass Spectrometry
BDE-209	Decabromodiphenyl ether
PCB	Polychlorinated biphenyls
DIN	Deutsches Institut für Normung
IVD	In vitro digestion model
UBM	Unified BARGE method
FOREhST	Fed ORganic Estimation Human Simulation Test
RBA	Relative bioavailability
